# Supplementary material for: Design of a patient-centered decision support tool when selecting an organ transplant center
Source: PLoS One. 2021 May 17;16(5):e0251102. doi: 10.1371/journal.pone.0251102 (PMC8128227; doi:10.1371/journal.pone.0251102)
Supplement: S1 Table — (DOCX) [file pone.0251102.s001.docx]

**Design of a patient-centered decision support tool when selecting an organ transplant center**

**Supplemental Materials: Tables**

| S1 Table: Full text of online survey questions and response options | |
| --- | --- |
| Question | **Response options** |
| Q1: What is your age? | [Text entry field] |
| Q2: What is your gender? | Male; Female |
| Q3: What is your highest level of education in years? | 9; 10; 11; 12 Completed High School; 13; 14; 15; 16 Completed Undergraduate degree; 17+ Graduate Studies |
| Q4: Have you ever needed or received an organ or other transplant? | Yes; No |
| Q5: Have any family members or friends ever needed or received an organ or other transplant? | Yes; No |
| Introduction text: The following page will display a small portion of a website specifically for patients who need an organ transplant, e.g. kidney or liver.  Even if you are not familiar with this procedure, your understanding of the information will be similar to many patients who are often faced with learning a great deal of complex, new information.  You will see a comparison of the quality of different transplant centers (a hospital that would treat these patients).  This would be one way you could learn about options and decide where to go.  Read this as if you were learning about a hospital for a medical procedure. | |
| [View randomly assigned image] | |
| Q6: Hypothetically, what hospital would you choose for a transplant based on the information on this list? | Lake Hospital; Alpine Hospital; Meadow Hospital |
| Q7: What was the most important factor in the decision? | Distance; Transplants in a Year; Survival On The Waitlist; Getting a Transplant Faster; One Year Organ Survival; No single factor was most important |
| Q8: Some transplant centers do not transplant living donors. This question is a test whether or not you are reading carefully. You must answer none of the above. | I do not believe living donor transplants would apply to me.; I believe the living donor transplants would apply to me.; I am not familiar with what these terms mean.; None of the above |
| Q9: Review the 1 year organ survival shown for Lake Hospital. Choose the option you believe is most accurate. | About 3 out of 5 patients have a transplanted organ that survives 1 year.; The 1 year organ survival at Lake Hospital is about the same as national rates.; Lake Hospital has about double the 1 year organ survival compared to Alpine Hospital.; Cannot determine from the information shown. |
| Q10: Briefly describe any information that you do not understand. If you understand everything, list something you would like to learn more about to help make a decision. | [Text entry field] |
| The images that follow will each display the same exact list of transplant centers in the same order.  The only difference is the style of the graphic icons used to display quality. | |
| [View all 6 images together] | |
| Q11: Select the style that you feel most clearly displays what option would be best. | Style A; Style B; Style C; Style D; Style E; Style F |
| Q12: Briefly describe why you selected this icon style. | [Text entry field] |
