# Supplementary material for: Design of a patient-centered decision support tool when selecting an organ transplant center
Source: PLoS One. 2021 May 17;16(5):e0251102. doi: 10.1371/journal.pone.0251102 (PMC8128227; doi:10.1371/journal.pone.0251102)
Supplement: S2 Table — (DOCX) [file pone.0251102.s002.docx]

**Design of a patient-centered decision support tool when selecting an organ transplant center**

**Supplemental Materials: Tables**

| **S2 Table**: Usability testing discussion guide questions. |
| --- |
| Part 1: Website navigation tasks |
| Task one  1. Read the information under “Centers That Transplant Patients Like You**”**  2. What do you think about the information (e.g. clear, I don’t understand)?  3. Click on the “Find Transplant Centers” button  Task two 1. Enter the required information at the two fields, please read out loud.  2. What do you think about the purpose of the required information?  3. Click on the “Patient Specific Custom Search” button  Task three 1. Enter the required information at the various fields, please read out loud.  *If pop up information is provided, ask patient if the information is clear.*  2. What do you think about the purpose of the required information?  3. Click on the “Find Transplant Centers” button  Task four  1. Read out loud the information on the screen that provides transplant centers search results.  2. Are the headings clear on top of the table?  3. What is the icon “I” represents? Is the information clear?  4. What are the two pictorial keys represent? Did you use them as references to interpret the results?  Task five  1. Navigate to the custom search heading,  See if patient use the mouse to roll over the “?”. If not, point that out.  2. Read the roll over information inside the orange box, is it clear?  3. Click on the selection box, can you explain what you are looking at?  4. What does the number represents in this column?  Task six  1.Based on the provided information, which hospital will you choose? Which criteria is the most important for you to make a decision? Why?  2. Is there any additional information you would like to see on this page that may help you to make a decision?    Task seven  1. Find the FAQ button  2. Read out loud the first two questions and answers. |
| Part 2: Website overview |
| 1. Please describe your overall impression on the navigation process of the overall website. 2. Do you think the navigation is unnecessarily complicated or easy to use? 3. Do you think there is any inconsistency on how you navigate through the site? 4. Please describe your impressions about the layout of the pages and what you think of the colors and graphics. 5. Do you think you would like to use this website again for the information that you are looking for? 6. What did you like best about the website? 7. What did you like least about the website? 8. If you were to describe your experience today to a friend, family member, or another patient in a sentence or two, what would you say? 9. What changes would you make to the website? What other information you would like to add? 10. Do you have any other final comments or questions? |
