# Supplementary material for: Design of a patient-centered decision support tool when selecting an organ transplant center
Source: PLoS One. 2021 May 17;16(5):e0251102. doi: 10.1371/journal.pone.0251102 (PMC8128227; doi:10.1371/journal.pone.0251102)
Supplement: S3 Table — (DOCX) [file pone.0251102.s003.docx]

**Design of a patient-centered decision support tool when selecting an organ transplant center**

**Supplemental Materials: Tables**

| **S3 Table**: Usability testing health history questionnaire for kidney transplant patients. Questionnaires for liver, heart, and lung patients were similar but included health questions specific to each organ type. |
| --- |
| What caused your own kidneys to stop working?  1) Diabetes  2) Hypertension  3) Polycystic kidney disease  4) Glomerular disease  5) Other: _______________________  6) Don’t know  Review this list of some medical problems. Please answer Yes or No whether a doctor or other health care provider has ever told you that you have these problems.  High Blood Pressure (Hypertension) Yes No  Diabetes Yes No  a. [If YES] Do you use insulin? Yes No  Asthma or Emphysema Yes No  Cancer Yes No  Stroke or Cerebrovascular accident (CVA) Yes No  High Cholesterol Yes No  Coronary Artery Disease or Heart Attack Yes No  Heart Failure Yes No  Irregular beating of the heart or Cardiac arrest Yes No  (heart stops beating)  Hepatitis or Liver Disease Yes No  Overweight Yes No  Have you had any of the following **during the past 1 year**?  Angina or Chest pains Yes No  Shortness of Breath Yes No  Have you **ever** had any of the following procedures?  Bypass heart surgery Yes No  Coronary Angioplasty (procedure to open clogged arteries in your heart)  Yes No  How many times have you been admitted to the hospital in the last 12 months?  Have you ever been evaluated for a kidney transplant at another transplant center?  Yes No Not Sure  If yes, ask: At which transplant center were you evaluated? ____________________  Are you on the list for a kidney transplant?  Yes No Not Sure  Table S3 Continued  Have you had a previous kidney transplant?  Yes No  What is your blood type?  O A B AB Not Sure  What is your height ‘ “ and weight ?  In general, how would you describe your health? Please use one of the following 5 terms.  1) Excellent  2) Very good  3) Good  4) Fair  5) Poor  Are you considering living kidney donation if you are approved for kidney transplant?  1) Yes  2) No |
