# Supplementary material for: Design of a patient-centered decision support tool when selecting an organ transplant center
Source: PLoS One. 2021 May 17;16(5):e0251102. doi: 10.1371/journal.pone.0251102 (PMC8128227; doi:10.1371/journal.pone.0251102)
Supplement: S4 Table — (DOCX) [file pone.0251102.s004.docx]

**Design of a patient-centered decision support tool when selecting an organ transplant center**

**Supplemental Materials: Tables**

| **S4 Table**: Selection of feedback from website users not enrolled in study. | |
| --- | --- |
| Response Type | Feedback |
| Patient seeking a transplant | This is much, much, much easier for a patient to use than the old system. |
| Transplant Recipient | The information provided on this website is necessary and essential to all patients and their families. The comparison data on each center is informative. The website gives patients a clear understanding of the important aspects of transplantation. |
| Transplant Recipient | Choosing a Transplant Center hinges upon considerations far more complex than what is presented here... The bigger issues in choosing a center are the expertise and stability not just of the surgeons, but if the entire staff supporting patients in the long period pre, during and post transplant, and the expertise of the Center in the many specialties relevant to what patients need..again before the Transplant and thereafter. Lastly, different Centers are more or less responsive to diverse populations, to engaging patients' families of partners, to enabling patients to continue or to return to work, to being proactive in helping patients deal with insurance and financial problems. |
| [Not listed] | I have kidney disease from cancer treatment and have a number of other medical issues. Not every center will list me - something I was unaware of until after I was fortunately listed at [my center]. If I had gone to another center and was refused I would have given up and not known I could try somewhere else. There should be a section with an explanation for patients like me, there are many of us that do not realize this. Thank you |
| Provider | This is a fantastic resource not only for patients but for those who are assisting patients in making a choice (payors included). |
